# Preparation and Evaluation of Adsorbents from Coal and *Irvingia gabonensis* Seed Shell for the Removal of Cd(II) and Pb(II) Ions from Aqueous Solutions

**DOI:** 10.3389/fchem.2017.00132

**Published:** 2018-01-26

**Authors:** Mercy A. Ezeokonkwo, Okechukwu F. Ofor, Julius U. Ani

**Affiliations:** Department of Pure and Industrial Chemistry, University of Nigeria, Nsukka, Nigeria

**Keywords:** lignite, adsorption, *Irvingia gabonensis*, isotherm, kinetics

## Abstract

Cd(II) and Pb(II) ions removal using adsorbents prepared from sub-bituminous coal, lignite, and a blend of coal and *Irvingia gabonensis* seed shells was investigated. Fourier transform infrared, scanning electron microscope and X-ray fluorescence analyses implicated hydroxyl, carbonyl, Al_2_O_3_, and SiO_2_ as being responsible for attaching the metal ions on the porous adsorbents. The optimum adsorption of carbonized lignite for the uptake of Cd(II) and Pb(II) ions from aqueous media were 80.93 and 87.85%, respectively. Batch adsorption was done by effect of adsorbent dosage, pH, contact time, temperature, particle size, and initial concentration. Equilibrium for the removal of Pb(II) and Cd(II) was established within 100 and 120 min respectively. Blending the lignite-derived adsorbent with *I. gabonensis* seed shell improved the performance significantly. More improvement was observed on modification of the blend using NaOH and H_3_PO_4_. Pb(II) was preferentially adsorbed than Cd(II) in all cases. Adsorption of Cd(II) and Pb(II) ions followed Langmuir isotherm. The adsorption kinetics was best described by pseudo-second order model. The potential for using a blend of coal and agricultural byproduct (*I. gabonensis* seed shell) was found a viable alternative for removal of toxic heavy metals from aqueous solutions.

## Introduction

The accumulation of toxic heavy metals in industrial wastewater effluents has become a great challenge in less developed countries such as Nigeria, as these effluents are indiscriminately discharged into water bodies or on land (Weber et al., 1991). Wastewater effluents generated in some chemical process industries contain cadmium, lead, copper, zinc, nickel, and chromium (Argun and Dursun, 2008), which if contained above a given concentration are hazardous and has the propensity of leading to fatal health challenges. In addition, these elements, unlike most pollutants are not ecologically degradable rather they undergo a universal ecological cycle in which soil and water are the main pathways (Nwokonkwo, 2008). High levels of heavy metals in soils could result in uptake by local and agronomic plants and leaching to ground and surface waters.

Sewage sludge application causes related problems like rise in injurious level of the heavy metal concentration in edible crops (Nwajei, 2005). Cadmium (II) can enter the food chain via plant uptake (Nwajei, 2005). Natural waters are contaminated by lead through several activities in industries, which include battery, paint, metal plating, smelting, textiles, and plastics, amongst others (Opeolu et al., 2008). All foods contain varying amounts of lead and cadmium. Concentrations of lead and selenium in plants may produce subsequent toxic effects (Nwajei, 2005). Cadmium and lead are among the most highly toxic metals commonly found in most industrial wastewater. Lead has been implicated as one of the three highly harmful heavy metals, which exhibit latent prolonged negative effect on human health; thereby resulting in a number of diseases such as hepatitis, anaemia, nephritic syndrome, and encephalopathy (Deng et al., 2006). It can also lead to brain and bone damage (Mishra, 2014). Cadmium on the other hand endangers human health as it can cause so many health disorder including kidney damage, diarrhea, itai—itai disease, mucous membrane destruction, bone damage, and vomiting. It also influences the formation of progesterone and testosterone (Johannes et al., 2006). These and many other health problems associated with lead and cadmium prompted the choice of lead and cadmium in this study. There is need for the concentration of heavy metals in industrial effluents to be controlled within tolerable levels (Meena et al., 2008) before being discharged into sewage systems, water ways, or on land to avoid ecological pollution.

Several technologies are available in literature for the removal of heavy metals from industrial wastewater (Anand et al., 1985; Kim et al., 1985; Murphy and Erkey, 1997; Monser and Adhoum, 2002; Erdem et al., 2004; Golder et al., 2007; Lin et al., 2008; Yuan et al., 2008), which include coagulation/flocculation, adsorption, reverse osmosis, and biological treatment. Of all these techniques, adsorption is the most efficient and versatile for removal of heavy metal. (Agarwal et al., 2006). Most of the other methods are non-selective and not very effective in the face of low metal concentration in the effluent (Mishra, 2014). According to Kurniawan et al. (2006), adsorption can be defined as “a mass transfer process by which a substance is transferred from the liquid phase to the surface of a solid, and becomes bound by physical and/or chemical interaction.” Adsorption occurs by transport of adsorbates from the bulk fluid to the adjoining fluid-adsorbent interface by film diffusion of adsorbates through the laminar boundary layer surrounding the adsorbent particle. This is followed by surface reaction of the adsorbate reactants to the internal surface of the porous adsorbent to form adsorbed products (Nabi et al., 2015). Several works on the adsorption of heavy metals using activated carbon prepared from various low-cost precursors have been reported (Kumar, 2006). In addition, the potential of coal and coal fly ash as adsorbents has been investigated (Gangoli et al., 1975; Grover and Narayanaswamy, 1982; Yadera et al., 1987; Moreno-Castilla et al., 1994; Martyniuk and Wieckowska, 1997; Menkiti and Onukwuli, 2011). Various research works have similarly been conducted on the use of blended adsorbents for wastewater effluent treatment (Panday et al., 1984; Nordiana and Siti, 2013). Nordiana and Siti (2013) reported that a blend of activated charcoal and peanut shell was more efficient in adsorbing lead ions from aqueous solution than the individual adsorbents. Wang and Xing (2002) also reported that phosphate-modified goethite improved both cadmium adsorption and adsorption process. Earlier studies have shown that adsorption efficiency increases with increase in carbonization temperature as well as the modification of the adsorbent with activating agents such as nitric acid (Kareem and Adisa, 2002), potassium permanganate (Muhammad et al., 2011), hydrochloric acid (Bada and Potgieter-Vermaak, 2008; Muhammad et al., 2011), hydrogen peroxide (Muhammad et al., 2011), and potassium hydroxide (Evbuomwan et al., 2013).

Despite the fact, that much work has been done on adsorption of heavy metals, there is still need for further exploitation of this area (Mishra, 2014). The use of cost effective, readily available, and ecologically friendly materials should be encouraged. There is need for low cost adsorbents with improved adsorption capacity and having little or no harmful effect to the ecosystem or environment. To this end, the focus of heavy metal adsorption studies, as demonstrated in this work, has shifted to the sourcing of adsorbents from natural products that are readily available such as coal, and some environmentally friendly agricultural byproducts namely ogbono (*Irvingia gabonensis*) seed shell, coconut shell, palm kernel shell, walnut shell, and almond shell (Golder et al., 2007). Coal is formed after long time of degradation of plant material in the ground. Coal is classified as lignite, sub-bituminous, bituminous, and anthracite depending (in that order) on the age of degradation of the plant matter that formed the coal, and the carbon content. According to statistical survey, there are proven coal reserves in the following parts of Nigeria: Anambra coal basin, covering 1.5 million hectares; Benue district (Omkpa-Ezimo), 175,000 hectares; Kogi district, 225,000 hectares; Enugu district, 270,000 hectares; Inyi deposit, south of Enugu city; the Afikpo deposit, Lafia Obi deposit, the Gombe deposit, and the Asaba lignite deposit (Odesola et al., 2013). Coal has many industrial applications which include electricity generation, metallurgical extraction of metals, and chemicals production. Little has been reported on the use of Nigerian coal for the preparation of adsorbent (Ani et al., 2012).

This study aims at evaluating the adsorption potential of different types of coal namely lignite and sub-bituminous coal, and *Irvingia gabonensis* seed shell (IGSS), for cadmium and lead removal. The objectives include finding a simpler and more versatile method for surface modification of coal to improve its morphology and surface area for increased adsorption rate.

## Materials and methods

### Reagents and instruments

Analytical grade of all the chemicals and reagents were used.

The instruments used include Mettler Toledo Seven compact pH meter, atomic absorption spectrometer (Buck Scientific, Model 210 VGP), Fourier transform infrared spectrophotometer (Model IRTracer-100 Shimadzu, Japan), electron scanning microscope (PHENOM PROX TESCAN, The Netherlands), and X-Ray fluorescence (Oxford Instruments, England). Others are heat treatment furnace (Kohaszati Gyarepito Vallat Budapest, type KCO−120), air-drying oven (BTOV 1423), Cisa cedaceria industrial electromagnetic Shaker (Model BA200N), digital water bath (Model DK600), Ohaus weighing balance (Model PA213), and a multifunctional oscillator.

### Sampling/sample preparation

Lignite was obtained from Garinmaiganga mine in Gombe State, sub-bituminous coal from Okaba mine in Kogi State; *Irvingia gabonensis* seed shells were obtained from Obe in Nkanu-west local government area of Enugu State, all in Nigeria. The samples were thoroughly washed, to remove extraneous materials such as dirt, sand and other impurities, and subsequently dried, and milled to fine particle sizes. They were then carbonized separately in a muffle furnace (Kohaszati Gyarepito Vallat Budapest, type KCO-120) at different temperatures.

### Preparation of adsorbate solution

The stock solutions of Cd(II) and Pb(II) ions of concentrations 1000 mg/L each were obtained by dissolving 2.03 g cadmium chloride, (CdCl_2_.2^1^/_2_H_2_O) and 1.6 g lead nitrate, [Pb(NO_3_)_2_], respectively with distilled water in a 1000 mL standard flask and made up to mark. The solutions were then diluted to desired working concentrations with distilled water.

### Preparation of adsorbents

The adsorbents were prepared by carbonization of the raw materials. For the first stage of the experiments, lignite, and sub-bituminous coal were carbonized at 800°C. This was done by measuring 700 g each of the coals into separate clay pots. The pots were covered, with the edges between the pots and their lids sealed with clay to prevent penetration of air. The samples were put in a muffle furnace and carbonized at 800°C for 2 h, following the method of Scientific Equipment Development Institute (SEDI), Enugu, Nigeria. Lignite, being the sample that had better adsorption efficiency was used in the determination of the effect of batch adsorption parameters, and was carbonized at different temperatures (400–1100°C) for the determination of the effects of carbonization temperature.

### Modification using *Irvingia gabonensis* seed shell

Lignite carbonized at 400°C, which gave the optimum percent yield of 75%, was blended with IGSS biomass in equal proportion. The mixing of the seed shell with lignite was made to find out if blending coal with agricultural byproduct would improve adsorption performance.

### Chemical modification of adsorbents

The adsorbents from lignite and its *IGSS* blend were chemically modified with 0.1 M of both NaOH and H_3_PO_4_ following the method of Argun and Dursun (2006). Twenty five grams of adsorbent was measured, washed severally with distilled water to remove particles sticking to the surface and any particle that could be soluble in water. The adsorbent was oven-dried at 85°C for 2 h. The sample was placed in a 500 mL conical flask containing 250 mL of the modifying reagent. It was then agitated at 200 rpm in a multifunctional oscillator for 4 h, left overnight and filtered to separate the adsorbent. The adsorbent was washed severally with distilled water to attain neutral pH. Finally, it was oven-dried at 85°C for 2 h and stored for use.

### Characterization of adsorbent

#### X-ray fluorescence analysis

The X-Ray Fluorescence (XRF) characterization was performed to determine the chemical compositions of the raw materials.

#### Fourier transform infrared analysis

Fourier transform infrared (FTIR) spectroscopic analysis was performed to give the vibration frequencies of the adsorbents lattice, which result from stretching of bending modes of the functional groups present in the activated carbon. The samples were examined in the range 400–4000 cm^−1^.The analysis was done using KBr as background material.

#### Scanning electron microscopy

The morphology of the prepared adsorbents was studied by use of scanning electron microscope (SEM). Little amounts of the prepared adsorbent samples were first put on a circular disc-like structure in the scanning electron microscope. Specific tapes were used to attach the samples to the surface of the disc. The disc was then positioned in the electron chamber, and the electron gun releases a beam of electrons used for the scanning. As the electrons interact with the atoms, images of the surface topography is produced, and viewed on a monitor. In order to obtain a more quality image and appropriate clarity, the magnification of the scan was adjusted. The specific magnifications images were later saved.

### Adsorption experiments

Four grams of the adsorbents prepared separately from lignite, sub-bituminous coal, and *IGSS*-coal blend were introduced separately to 100 mL of 300 mg/L metal ion solution in a conical flask and agitated on the multifunctional oscillator set at 200 rpm for 2 h at room temperature. After adsorption was completed, the solution was filtered using Whatman No. 1 filter paper. The residual metal ion concentrations of the solution were determined by atomic absorption spectrophotometer. The adsorption capacity and efficiency were calculated using Equations (1, 2), respectively:

(1) $$\begin{array}{r} q_{e}=\frac{\left(C_{i}-C_{e}\right)V}{m} \end{array}$$

(2) $$\begin{array}{r} \%Adsorption=\frac{C_{i}-C_{t}}{C_{i}}x100 \end{array}$$

Where *q*_*e*_ is the amount of metal adsorbed at equilibrium (mg/g),

m is the mass of adsorbent (g),

*C*_*i*_ and *C*_*e*_ are the initial and equilibrium concentrations of the metal ions (mg/L), while *C*_*t*_ is the concentration at time t (minutes).

Comparative adsorption experiment was carried out using adsorbents from lignite and sub-bituminous coal. Dosage of adsorbent from lignite was varied in the range 5–40 g/L to determine its effect on adsorption of the metal ions. The effect of pH on the adsorption was carried out by adjusting the metal solution pH from 4 to 10, using 0.1 M HCl and 0.1 M H_3_PO_4_.

The effect of contact time on adsorption was determined by varying time of the process in the range 20–120 min. To study the effect of temperature the flask was agitated at a temperature that is within the range of 30–65°C.

Carbonized unmodified lignite was used for adsorption at different particle sizes (250, 355, 500, 710, 850, and 1400 μm) to determine effect of particle size. The effect of initial metal ion concentration was determined by varying the adsorbate concentration from 100 to 350 mg/L.

## Results and discussion

### XRF, SEM, and FTIR techniques

#### X-ray fluorescence characterization

The XRF characterization was performed to obtain the chemical compositions of the raw materials (lignite, sub-bituminous coal, and *Irvingia gabonensis* seed shell). These are as given in Table 1. It could be observed that Al_2_O_3_ and SiO_2_ are the key constituents of these materials. Fe_2_O_3_ is present in considerable amount, while Na_2_O, MgO, P_2_O_5_, K_2_O, CaO, and TiO_2_ are present in trace quantities. Hence Al_2_O_3_, SiO_2_, and Fe_2_O_3_ might have contributed in the removal of Cd(II) and Pb(II) ions from the simulated waste water.

**Table 1:** XRF Results of lignite, sub-bituminous coal, and *Irvingia gabonensis* seed shell.

**Chemical constituent**	**Raw material composition (Wt %)**		
	**Lignite**	**SBC**	**IGSS**
Na_2_O	1.979	2.835	2.842
MgO	2.235	1.164	1.726
Al_2_O_3_	19.475	19.686	20.374
SiO_2_	17.408	17.467	11.520
P_2_O_5_	1.100	1.105	1.681
K_2_O	0.266	0.285	3.000
CaO	4.704	0.665	2.356
TiO_2_	0.773	0.852	0.596
Fe_2_O_3_	6.278	5.986	6.503

#### Scanning electron microscopy

**Figure 1:**
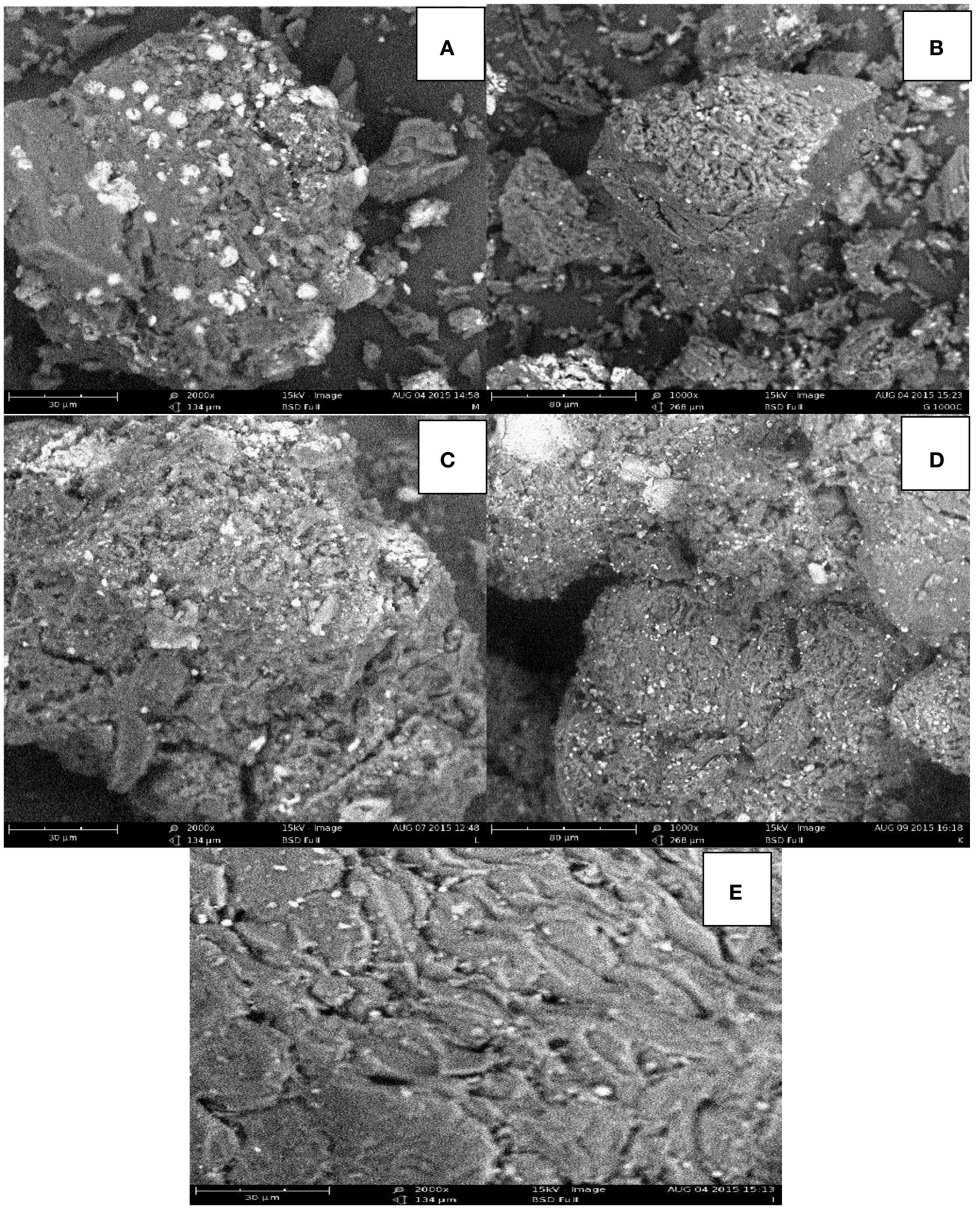
SEM images of **(A)** carbonized unmodified sub-bituminous coal **(B)** carbonized unmodified lignite **(C)** spent carbonized unmodified lignite after Cd(II) adsorption **(D)** spent carbonized unmodified lignite after Pb(II) adsorption **(E)** Irvin*gia gabonensis* seed shell lignite blend.

The SEM images of the experimental adsorbents are shown in Figure 1. The adsorbents have irregular and porous surface structure, which is a characteristic of a potential adsorbent. The porous structure is an indication that physical adsorption has serious effect on the removal of the lead and cadmium ions from aqueous solution (Vafakhah et al., 2014). It is evident that the carbon particles have wide range of pore sizes. There appears to be accumulated deposits in the pores of the spent adsorbents probably due to adsorption of the metal ions onto the pores on the surface of the adsorbents.

#### Analyses of the FTIR spectra

**Figure 2:**
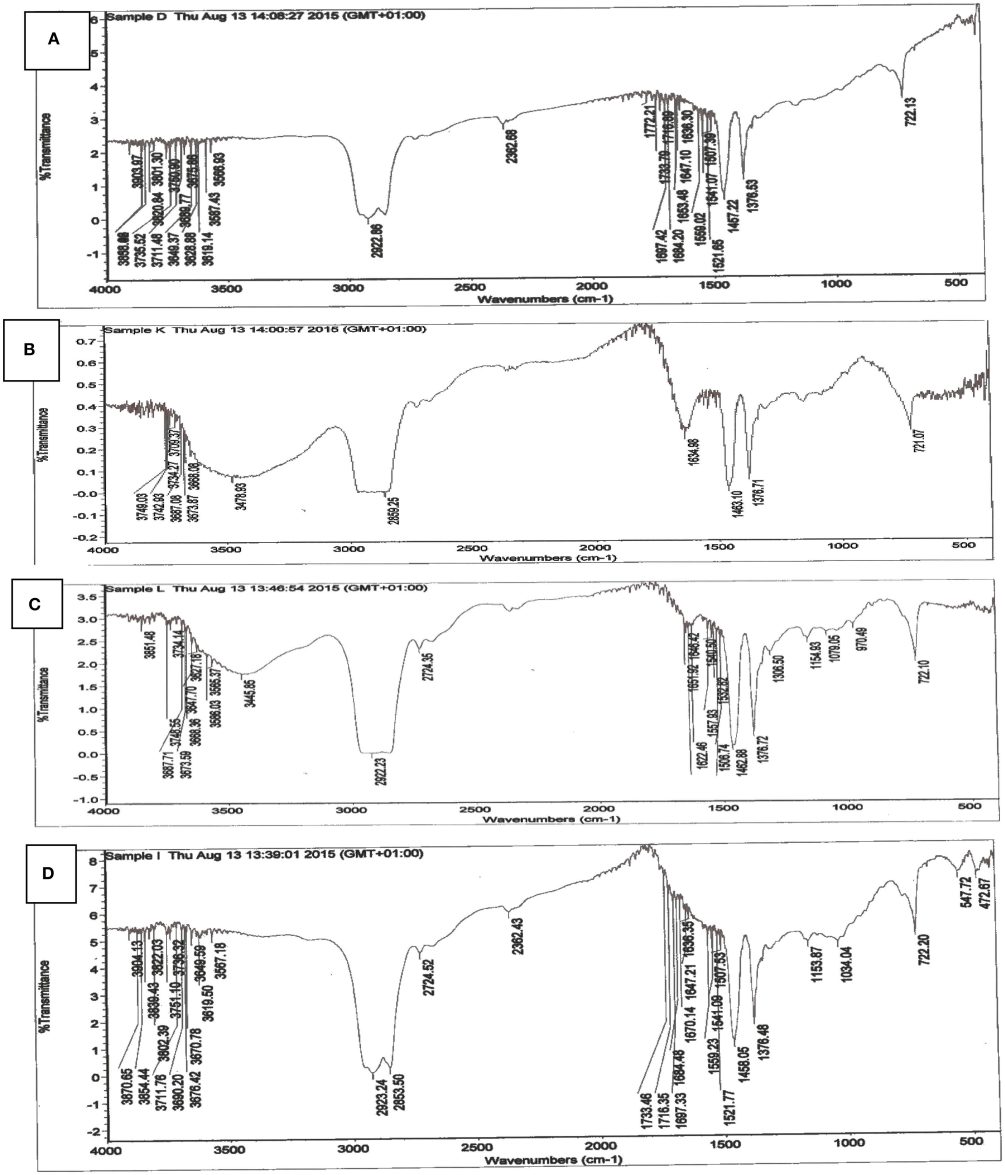
FTIR spectrum of **(A)** carbonized unmodified lignite **(B)** spent carbonized unmodified lignite after Cd(II) adsorption **(C)** spent carbonized unmodified lignite after Pb(II) adsorption **(D)** *Irvingia gabonensis* seed shell lignite blend.

FTIR spectrum of carbonized unmodified lignite (CUL) is shown in Figure 2A. The figure indicates the presence of majorly carbonyl and hydroxyl groups, which constitute important adsorption centres. By comparing the FTIR spectrum of CUL and spent CUL (Figures 2A,B), changes in peaks were observed. These changes may be due to interaction between the functional groups and metal ions because of adsorption or chemical reaction. The sharp peaks at 3567, 3676, 3690, 3751, 3649, 3821, 3712, 3736, 3858, and 3904 cm^−1^ were due to O-H stretching vibrations in alcohols and phenols. Whereas, the peaks at 3587, 3619, and 3629 cm^−1^ are assigned to oximes O-H stretching vibrations. There is absorption band at 2923 cm^−1^ present in the CUL, which can be attributed to the presence of aliphatic CH_3_. This band was observed in the spent CUL at 2950 cm^−1^ (Figure 2B). A peak is also observed at 2860 cm^−1^ in the CUL. The corresponding peak is observed at 2858 cm^−1^ in the spent CUL. These peaks are assigned to the aliphatic CH_2_ group. The peaks 1734 and 1772 cm^−1^ are assigned to C = O stretching vibrations of aldehydes and ketones. The peak at 1697 is assigned to C = O stretching vibrations in α-amino acids whereas the peak at 1684 is due to the C = O stretching vibrations in α, β-unsaturated acids.

Most of these peaks are not seen in the FTIR spectra of the spent carbonized lignite used to remove cadmium (Figure 2B) and lead (Figure 2C). The reason for this could be the interaction between the functional groups and metal ions as a result of adsorption. This is an indication that binding of metal ions on the adsorbent took place.

The infrared spectrum of *Irvingia gabonensis* seed shell lignite blend (IGSSLB) is shown in Figure 2D. Analyses of FTIR spectra of IGSSLB revealed a sharp and high intensity at 2923 cm^−1^ ascribed to C-H stretching vibration of alkanes in the compound. The peak range that centres around 2362–1376 cm^−1^ characterize: O-H, N-H, and C-H stretching vibrations. Absorption signal at 722 cm^−1^ reveal the presence of aromatic C-H out of plane blend, skeletal C-C vibrations. The frequencies and the proposed assignments of vibrations are based on previous assignments.

### Effect of process parameters on batch adsorption

#### Effect of carbonization temperature

**Figure 3:**
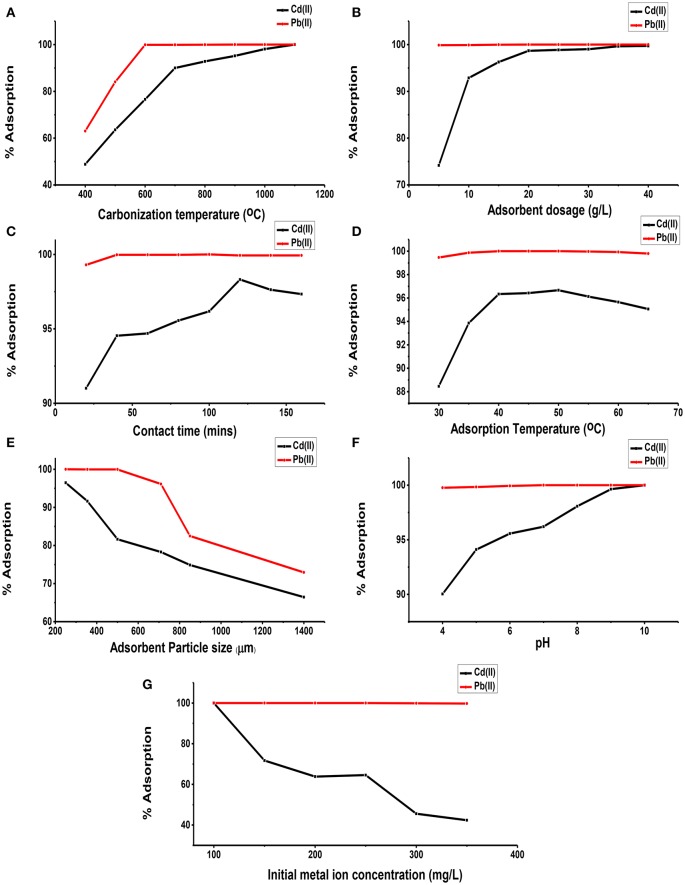
Effect of carbonization temperature **(A)**, adsorbent dosage **(B)**, contact time **(C)**, adsorption temperature **(D)**, adsorbent particle size **(E)**, pH **(F)**, and initial metal ion concentration **(G)** on the adsorption of Cd(II) and Pb(II) on carbonized unmodified lignite adsorbent samples.

Figure 3A shows the effect of the carbonization temperature of unmodified lignite on Cd(II) and Pb(II) adsorption from aqueous solutions. The adsorption of both Cd(II) and Pb(II) ions by CUL increased with increase in carbonization temperature until the adsorption reached 100%. This can be as a result of the increased adsorption properties of the adsorbents at higher temperatures as more volatile matter is released at the higher temperatures allowing for the creation of more pores unto which more metal ions are adsorbed. The Cd(II) adsorption was more gradual before peaking at 100%. Ozer et al. (1998) has reported a similar trend for the adsorption of cadmium on activated carbon from sugar beet pulp. Also, Figure 3A shows that Pb(II) ions adsorption was faster to reach the peak of 100% and remained constant. Thus, it was shown that Pb(II) was more preferentially adsorbed than Cd(II). Similar result has been reported by Kongsuwan et al. (2006).

#### Effect of adsorbent dosage and contact time

The dependence of Cd(II) and Pb(II) adsorption on adsorbent dosage was determined by varying the amount of adsorbents from 5 to 40 g/L, while keeping other parameters constant. Figure 3B shows the adsorption of Cd(II) and Pb(II) efficiencies for the different dosages used. It can be observed that in general, adsorption efficiencies of the adsorbent increased with increase in dosage. The percentage removal of Pb(II) increased from 99.87% at adsorbent dosage of 5 g/L to 100% at adsorbent dosage of 20 g/L, and remained constant till 40 g/L adsorbent dosage. For Cd(II), percentage adsorption increased from 74.17% at 5 g/L to 99.70% at 40 g/L. The increased adsorption pattern for both metal ions could be attributed to higher number of available adsorption sites as dosage increases. This agrees with the observations by Kumar et al. (2012) and Gala and Sanak-Rydlewska (2011).

In order to determine the time required for equilibrium adsorption of metal ions, the effect of contact time on the adsorption of Cd(II) and Pb(II) ions from aqueous solution on the surface of CUL was investigated over a period of time. In all the cases, increased adsorption of metals with increasing contact time was observed until equilibrium, after which there was slight decrease in efficiency, as shown in Figure 3C. For Cd(II) ion, equilibrium was reached at 120 min at 98.31%. Afterwards the adsorption efficiencies decreased to 97.63 and 97.33% at 140 and 160 min respectively. However, for the Pb(II) ion, adsorption increased from 99.30 to 99.97% at the early stage, remained constant until peaking at 100 min at 100% before dropping to 99.93% at 120 min and then remaining constant. This phenomenon can be due to the fact that, initially, all the active sites on the CUL were vacant and were used for initial adsorption; subsequently, as time increased, the adsorption rate of metal ions became constant at equilibrium due to saturation of active sites in agreement with a work reported elsewhere (Meitei and Prasad, 2013).

#### Effect of adsorption temperature

Figure 3D shows the effect of solution temperature on the adsorption of Cd(II) and Pb(II) ions on carbonized unmodified lignite. As shown in Figure 3D, efficiency of Cd(II) increased from 88.45% at 30°C to 96.67% at 50°C. Beyond 50°C, it decreased. The same trend was observed in the adsorption of Pb(II) on CUL. Similar results have been reported elsewhere (García-Rosales and Colín-Cruz, 2010; Park et al., 2010; Giri et al., 2012). The increase in adsorption of Cd(II) and Pb(II) ions on carbonized unmodified lignite with the increase in temperature showed that the adsorption process is endothermic, which may be due to the higher rate of diffusion of the cations onto the adsorbent's particle surface at moderately higher temperatures. The high surface diffusion is due to the higher random motion of the adsorbates, resulting from the rise in thermal energy. As the temperatures were increased beyond the optimum values [50°C for Cd(II) and 40°C for Pb(II)], the adsorbent surface might decompose leading to reduced adsorption capacity as explained by Nabi et al. (2015). Table 2 gives the thermodynamic data (plot not shown) of the adsorption of Cd(II) by carbonized unmodified lignite. The Gibbs free energy change (ΔG) increased with increase in temperature. The positive values of the enthalpy change (ΔH) and (ΔG) indicate that the process is endothermic and thermodynamically non-spontaneous. The value of ΔH (Table 2) is +1027.78 J/mol indicating chemical adsorption. The reason that the adsorption process involves a decrease in entropy might be because a molecule in solution has more freedom of motion than one that is attached to a surface which implies decreased disorderliness on the adsorbent surface.

**Table 2:** Thermodynamic data for adsorption of Cd(II) by carbonized unmodified lignite.

**Temperature (K)**	**ΔG (J/mol)**
303	4,318.36
308	4,372.66
313	4,426.96
318	4,481.26
323	4,535.56
328	4,589.86
333	4,644.16
338	4,698.46
**ΔH (J/mol)**	**–ΔS (J/mol K)**
1027.78	10.86

#### Effect of adsorbent particle size

The influence of particle size of CUL on the adsorption of Cd(II) and Pb(II) ions from aqueous solution is shown in Figure 3E. For Cd, adsorption efficiency was highest (96.43%) at 250 μm, being the least particle size, while the lowest efficiency (66.43%) was recorded at 1400 μm, being the largest particle size. Also for Pb, at 250 μm adsorption efficiency was 100%, while at 1400 μm, it became 72.92%. A close observation of the results shows that efficiency is inversely related to particle size. As the particle size decreased, both surface area and adsorption efficiency of the adsorbent increased. Therefore, a decrease in adsorption with increasing particle size is due to a decrease in the surface area of the adsorbent, consistent with results reported by other authors (Badmus et al., 2007; Ozer et al., 2007; Kannan and Veemaraj, 2010; Banerjee et al., 2012; Kelly-Vargas et al., 2012; Barka et al., 2013).

#### Effect of adsorption solution pH

The pH of the solution has a considerable effect on the removal of heavy metals from aqueous solutions, because the surface charge of the adsorbent and the degree of ionization and speciation of the adsorbate are controlled by pH (Park et al., 2010). Figure 3F shows the effect of pH on the adsorption of Cd(II) and Pb(II) ions. The adsorption of Cd(II) was from under 90% while that of Pb(II) ions was from above 99%. The optimum adsorption for Cd(II) was at pH 10 while that of Pb(II) was at pH 6.5. However, percentage adsorption increased as pH increased. This could be attributed to the fact that as the pH of solution increases, the adsorptive removal of cationic metals increases, whereas that of anions decreases. At lower pH, the overall surface charge of the adsorbent may be positive. The H^+^ ions competed effectively with the Cd(II) and Pb(II) ions, causing a decrease in adsorption capacity. When pH values were increased, the CUL surface became increasingly negatively charged, which favoured the removal of metal ions as a result of electrostatic interaction. This is similar to the observations made by Njoku et al. (2011) and Taha et al. (2011).

The differential removal of the two ions may be attributed to the difference in their ionic radius. It has been shown that the smaller the ionic radius or area, the greater is its tendency to be hydrolyzed leading to reduced sorption (Horsfall and Spiff, 2005).

#### Effect of initial metal ion concentration and adsorbent modification

Figure 3G shows that increase in the initial metal concentration led to a decrease in removal efficiency. There was 100% removal of Pb(II) ions at concentrations 100–250 mg/L, whereas at concentrations 300 and 350 mg/L adsorption of 99.83 and 99.76% were respectively obtained. However, in the adsorption of Cd(II), there was a proportionate decrease in removal efficiency as the initial metal concentrations were increased. This observation could be due to the saturation of adsorption sites on CUL surface. This is in agreement with the results reported by previous workers (Boota et al., 2009; Kannan and Veemaraj, 2010; Sahmoune et al., 2011; Taha et al., 2011; Kumar et al., 2012). By applying 300 mg/L initial concentration (C_i_) of the metal ions (Cd and Pb) adsorption, 100% removal was recorded for each. However, when the concentrations were increased to 400 mg/L for ions (Cd and Pb), 80.93 and 87.85% removal were respectively obtained. Thus, carbonized unmodified lignite is a potentially good adsorbent for the removal of Cd and Pb ions from aqueous solution. Adsorption of Cd(II) and Pb(II) ions on adsorbent from lignite and sub-bituminous coal were compared at initial metals [Cd(II) and Pb(II)] concentration of 1000 mg/L. The adsorption for Pb(II) was 192 mg/L (19.2%) and 294.90 mg/L (29.49%) on sub-bituminous coal and lignite respectively. On the other hand, amount adsorbed for Cd(II) was 142.85 mg/L (14.29%) and 268.00 (26.80%) on sub-bituminous coal and lignite respectively. it could be observed that the amount adsorbed for all was not up to 300 mg/L. As a result, 300 mg/L was chosen as the working concentration for subsequent experiments, lignite on the other hand was used having more adsorption capacity than sub-bituminous coal.

Blends of coal were prepared by mixing lignite carbonized at 400°C with *Irvingia gabonensis* seed shell, NaOH and H_3_PO_4_ in order to achieve improved adsorption efficiency. Table 2 presents how various modifications of the carbonized lignite affected the adsorption efficiencies of the adsorbent. Upon modification of the CUL, the performance was significantly improved. For example, in Cd(II) adsorption, the sodium hydroxide and phosphoric acid modified lignite (SHML and PAML) increased adsorption efficiencies from 48.8 to 64.17% and 63.70% respectively. The Pb(II) adsorption was similarly enhanced due to the chemical modification (using NaOH and H_3_PO_4_) of the adsorbents.

In a work, Argun and Dursun (2006) made similar observations. There was also a similar trend of increased adsorption efficiencies with the blended derivatives namely *Irvingia gabonensis* seed shell lignite blend (IGSSLB), phosphoric acid modified *Irvingia gabonensis* seed shell lignite blend (PAMIGSSLB) and sodium hydroxide modified *Irvingia gabonensis* seed shell lignite blend (SHMIGSSLB) as given in Table 3. However, adsorbents modified with NaOH had better efficiencies than those modified with H_3_PO_4_. Highest efficiency was obtained with sodium hydroxide modified SHMIGSSLB.

**Table 3:** Effect of adsorbent modification on Cd(II) and Pb(II) Adsorption [C_i_ of Cd(II) and Pb(II) = 300 mg/L].

**Sample**	**C**_**e**_ **(mg/L)**		**q**_**e**_ **(mg/g)**		**% Adsorption**		**Surface area (m**^**2**^**/g)**	
	**Cd**	**Pb**	**Cd**	**Pb**	**Cd**	**Pb**	**Cd**	**Pb**
CUL (400°C)	153.60	111.0	3.66	4.73	48.80	63.00	805.80	865.80
PAML	108.90	47.0	4.78	6.33	63.79	84.30	857.30	887.30
SHML	107.50	46.2	4.81	6.35	64.17	84.60	888.90	888.90
IG SSLB	106.30	45.5	4.84	6.36	64.57	84.83	889.40	888.10
PAMIGSSLB	82.90	10.3	5.43	7.24	72.37	96.57	893.90	890.50
SHMIGSSLB	80.00	10.0	5.50	7.25	73.33	96.67	895.70	891.90

### Adsorption isotherm studies

Some routinely used adsorption isotherm models were applied to describe the adsorption mechanism involving carbonized unmodified lignite. The experimental data at 25°C were fitted into the following linear adsorption isotherm equations representing the selected isotherm models:

(3) $$\begin{array}{r} \left(\frac{C_{e}}{q_{e}}\right)=\left(\frac{1}{K_{L}q_{m}}\right)+\left(\frac{C_{e}}{q_{m}}\right) \end{array}$$

(4) $$\begin{array}{r} \ln\;q_{e}=\ln\;K_{f}{+}^{1}{/}_{n}\ln\;C_{e} \end{array}$$

(5) $$\begin{array}{r} q_{e}=B_{T}\ln\;A_{T}+B_{T}\ln\;C_{e} \end{array}$$

where Equations (3–5) represent Langmuir, Freundlich, and Temkin adsorption isotherms. Symbols contained in the adsorption isotherm equations are: the maximum monolayer adsorption capacity (*q*_*m*_), and adsorption constants (*K*_*L*_, *K*_*f*_, *B*_*T*_, *A*_*T*_, and 1/n).

**Figure 4:**
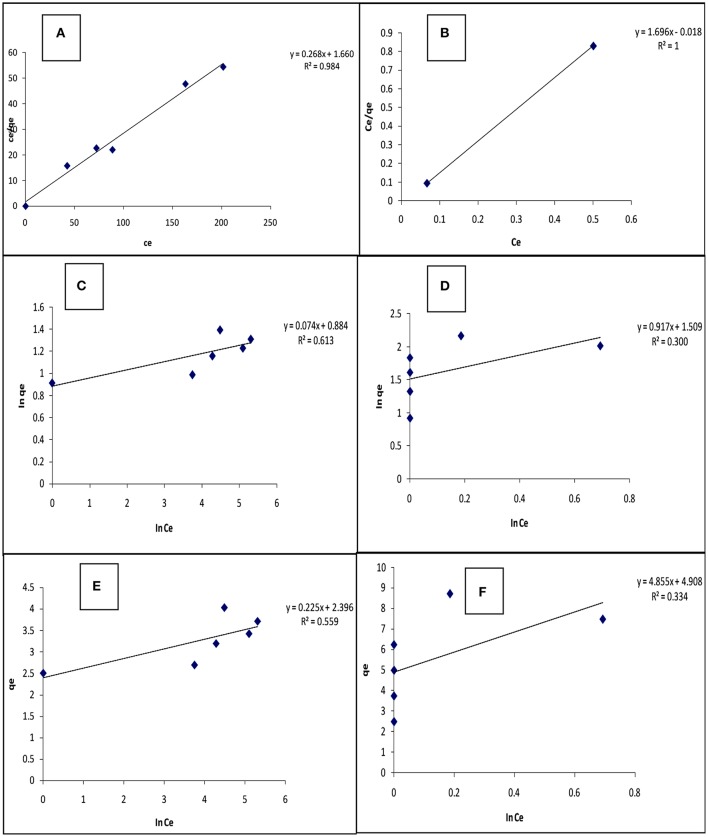
Langmuir **(A,B)**; Freundlich **(C,D)**; and Temkin **(E,F)** isotherms for the adsorption of Cd(II) and Pb(II) respectively, on carbonized unmodified lignite at 25°C.

The equilibrium isotherm parameters for this study were obtained from the slopes and intercepts of Equations (3–5) generated from their respective plots (Figure 4) and given in Table 4. The values of the maximum monolayer adsorption capacity of the adsorbent (*q*_*m*_) and the Langmuir adsorption equilibrium constant (*K*_*L*_) can be determined from the intercept and slope respectively of the linear plot of $\left(\frac{C_{e}}{q_{e}}\right)$ vs. *C*_*e*_. The Linearized Langmuir adsorption isotherm was developed with slope, intercept and regression coefficients equal 0.268, 1.660, and 0.984 respectively for Cd(II), and 1.696, −0.018, and 1.000 respectively for Pb(II). The Langmuir constants were calculated from intercept and slope [*q*_*m*_ = 3.731 (mg/g) (Cd), 0.590 (mg/g) (Pb) and *K*_*L*_ = 0.162 L/mg (Cd), −94.162 L/mg (Pb)].

**Table 4:** Adsorption isotherm data for carbonized unmodified lignite at 25°C.

**Isotherm Model**	**Cd(II)**	**Pb(II)**
**LANGMUIR**		
q_m_ (mg/g)	3.731	0.590
K_L_ (L/mg)	0.162	−94.162
*R*^2^	0.984	0.992
R_L_	0.017	0.0000303
**FREUNDLICH**		
K_f_ (L/g)	7.656	32.285
1/n	0.074	0.197
*R*^2^	0.613	0.300
**TEMKIN**		
*A*_*T*_(L/g)	4.46 x 10^10^	10.255
*B*_*T*_(mg/g)	0.225	4.855
*R*^2^	0.559	0.334

The values of *K*_*f*_ [Freundlich constant related to adsorption (mg/g)] and ^1^/_n_ (Freundlich constant related to the intensity of adsorption) can be determined from the intercept and slope, respectively, of linear plot of ln *q*_*e*_ vs. ln *C*_*e*_. The linearized Freundlich adsorption isotherm was developed with slope, intercept and regression coefficients equal to 0.074, 0.884, and 0.613 respectively for Cd(II), and 0.917, 1.509, and 0.300 respectively for Pb(II). The Freundlich constants were calculated from intercept and slope [*K*_*f*_ = 7.656 L/g (Cd), 32.285 L/g (Pb), and 1/n = 0.074 (Cd), 0.197 (Pb)]. Freundlich constant 1/n was < 1 in both Cd and Pb adsorption, indicating that the adsorbent surface is heterogeneous.

The values of *B*_*T*_ [constant related to the heat of adsorption (mg/g)] and *A*_*T*_ [Temkin isotherm constant (L/g)] can be determined from the intercept and slope, respectively, of linear plot of *q*_*e*_ vs. ln *C*_*e*_. The linearized Temkin adsorption isotherm was developed with slope, intercept, and regression coefficients equal 0.225, 2.396, and 0.559 respectively for Cd(II), and 4.855, 4.908, and 0.334 respectively for Pb(II). The Temkin constants were calculated from intercept and slope [*B*_*T*_ = 0.225 mg/g (Cd), 4.885 mg/g (Pb), and *A*_*T*_ = 4.46 × 10^10^ L/g (Cd), 10.255 L/g (Pb)].

Because the regression coefficients (*R*^2^) for the Langmuir, Freundlich, and Temkin isotherms are (Cd): 0.984, 0.613, and 0.559, and (Pb): 0.992, 0.300, and 0.334 respectively, it appears that the Langmuir isotherm correlates the adsorption data better than the other isotherms. This phenomenon suggests that the adsorption process may be attributed to a monolayer adsorption. The R_L_ (Langmuir isotherm separation factor) values obtained for Cd(II) and Pb(II) ions respectively, are 1.7 × 10^−2^ and 3.03 × 10^−5^ suggesting that the adsorption of the metals unto the adsorbent surface was favourable (0 < R_L_ < 1). (Jumina et al., 2007) defined R_L_ as:

(6) $$\begin{array}{r} R_{L}=1/\left(1+KC_{o}\right) \end{array}$$

where *K* is the adsorption constant and *C*_*o*_ is the initial concentration of adsorbate (g/L). Further, the lower the value of *R*_*L*_ is indicative of a more favourable adsorption.

### Adsorption kinetics

Adsorption kinetics study was performed by varying time of the process for Cd(II) and Pb(II) ions removal using carbonized unmodified lignite, in the range 20–120 min. In all the cases, increased adsorption of metals with increasing contact time was observed until equilibrium, after which there was slight decrease in efficiency, as shown in Figure 3C. The adsorption got to equilibrium because at that point the metals could not be removed anymore by the adsorbent.

The kinetic data obtained for the adsorption of Cd(II) and Pb(II) ions are fitted by pseudo first-order kinetic model, pseudo second-order kinetic model, and intra-particle diffusion model represented by Equations (6–8), respectively.

(7) $$\begin{array}{r} \ln\;\left(q_{e}-q_{t}\right)=\ln\;q_{e}-k_{1}t \end{array}$$

(8) $$\begin{array}{r} \frac{t}{q_{t}}=\frac{1}{k_{2}q_{e}^{2}}+\frac{t}{q_{e}} \end{array}$$

(9) $$\begin{array}{r} q_{t}=k_{i}t^{0.5}+I \end{array}$$

**Figure 5:**
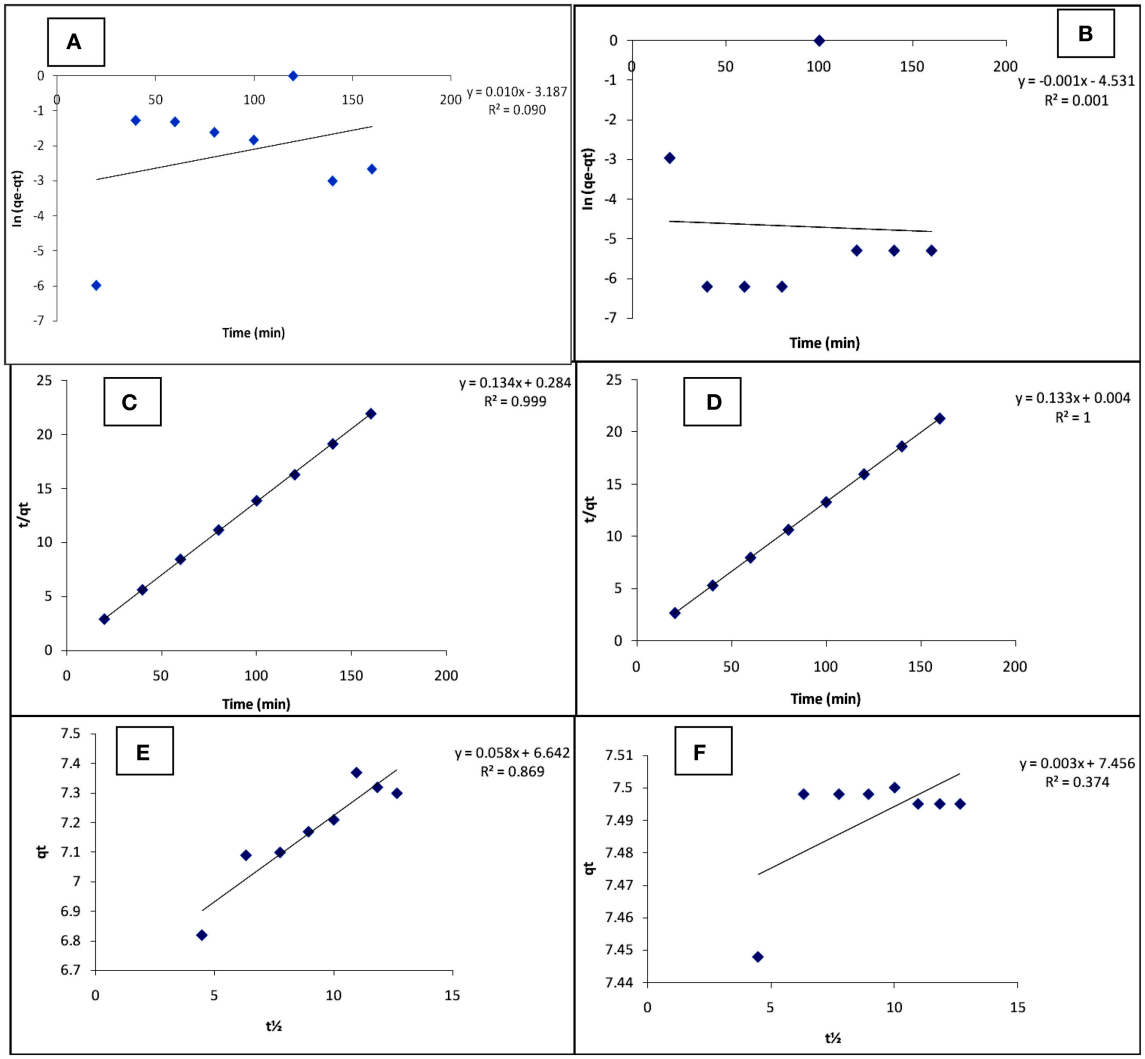
Pseudo first order **(A,B)**; Pseudo second order **(C,D)**; and Intra particle diffusion **(E,F)** for the adsorption of Cd(II) and Pb(II) respectively, on carbonized unmodified lignite at 25°C.

The adsorption kinetic parameters for the present study were obtained from the slopes and intercepts of Equations (6–8) generated from their respective plots (Figure 5) and given in Table 5. The values of the calculated equilibrium adsorption capacity (*q*_*e*_) and the rate constant for pseudo first-order model (*k*_1_) can be determined from the intercept and slope respectively of the linear plot of ln (*q*_*e*_ − *q*_*t*_) vs. *t*(min). The pseudo first-order kinetic model was developed with slope, intercept and regression coefficients equal 0.010, −3.187, and 0.090 respectively for Cd(II), and −0.001, −4.531, and 0.001 respectively for Pb(II). The pseudo first- order parameters were calculated from intercept and slope [*q*_*e*_ = 6.5 × 10^−4^ (mg/g) (Cd), 2.94 × 10^−4^ (mg/g) (Pb) and *k*_1_ = 2.303 × 10^−2^ /min (Cd), −2.300 × 10^−3^ /min (Pb)].

**Table 5:** Chemical kinetic data of carbonized unmodified lignite at 25°C.

**Model/Parameters**	**Cd(II)**	**Pb(II)**
**PFO**		
q_e_cal. (mg/g)	6.5 × 10^−4^	2.94 × 10^−5^
k_1_(/min)	2.303 × 10^−2^	−2.3 × 10^−3^
*R*^2^	0.090	0.001
q_e_exp.(mg/g)	7.37	7.50
**PSO**		
q_e_cal. (mg/g)	7.46	7.52
k_2_ (g/mg min)	6.33 × 10^−2^	4.42
*R*^2^	0.999	1
q_e_exp. (mg/g)	7.37	7.50
**IPD**		
K_d_(mg/g ${\text{min}}^{\frac{1}{2}}$)	0.058	0.003
C	6.642	7.456
*R*^2^	0.869	0.374

The values of the calculated equilibrium adsorption capacity (*q*_*e*_) and the rate constant for pseudo second-order model (*k*_2_) can be determined from the intercept and slope respectively of the linear plot of $\frac{t}{q_{t}}$ vs. *t*(min). The pseudo second-order kinetic model was developed with slope, intercept and regression coefficients equal 0.134, 0.284, and 0.999 respectively for Cd(II), and 0.133, 0.004, and 1.000 respectively for Pb(II). The pseudo second-order parameters were calculated from intercept and slope [*q*_*e*_ = 7.46 (mg/g) (Cd), 7.52 (mg/g) (Pb), and *k*_2_ = 6.33 × 10^−2^ g/mg min (Cd), 4.42 g/mg min (Pb)].

The regression coefficient (*R*^2^) values of the adsorption process (Table 4) indicated better agreement with the pseudo-second order (PSO) model [0.999 (Cd) and 1.000 (Pb)] than the pseudo-first order (PFO) model [0.090 (Cd), 0.001 (Pb)], for the metal ions removal. In addition, the calculated equilibrium adsorption capacity (q_e_cal.) values for both metals [7.46 (mg/g) (Cd) and 7.52 (mg/g) (Pb)] are quite close to the experimental equilibrium adsorption capacity (q_e_exp.) values (7.37 (mg/g) (Cd) and 7.50 (mg/g) (Pb) in the pseudo-second order model, suggesting that adsorption followed the pseudo-second order model. The calculated q_e_ [6.5 × 10^−4^ (mg/g) (Cd) and 2.94 × 10^−5^ (mg/g) (Pb)] and the experimental q_e_ values are wide apart in the pseudo-first order model. The rate constant (*k*_2_) values for PSO are significantly higher than those (*k*_1_ values) for PFO which further supports that the adsorption favours the pseudo second-order kinetic model.

The kinetic data was further analyzed with the intra-particle diffusion (IPD) model. A linear plot of *q*_*t*_ vs. t^1/2^ was used to obtain the constants K_d_ and C. Intra-particle diffusion is the sole rate-controlling step if the plot is linear and passes through the origin (C = 0). The *R*^2^ value obtained for the two metal ions indicates the existence of an intra-particle diffusion mechanism, more in Cd(II) ion (0.869) removal than in Pb(II) ion (0.374) adsorption, although it is not the sole rate-controlling step (C≠0). Occurrence of the intercept C shows the existence of a boundary layer effect, indicating a surface phenomenon such as mass transfer or liquid film diffusion in the adsorption process.

## Conclusions

In this work, lignite and sub-bituminous coal with blends and modifications were explored as precursors for the removal of toxic pollutants from wastewater effluents. Lignite was found to be more efficient than sub-bituminous coal in the adsorption of Cd(II) and Pb(II) from aqueous media. The carbonized unmodified lignite was found to perform optimally at pH 10, contact time 120 min, adsorption temperature 50°C, adsorbent particle size of 250 μm and dosage of 40 g/L. FTIR showed that hydroxyl and carbonyl groups may be the major functional groups responsible for the binding of the metal ions on the prepared absorbents. X-ray refractive fluorescence showed that Al_2_O_3_ and SiO_2_ were the major constituents of the prepared adsorbents. Lignite and *Irvingia gabonensis* seed shell performed better when modified with NaOH and H_3_PO_4_. For Cd(II) adsorption efficiency, the sodium hydroxide and phosphoric acid modified lignite (SHML and PAML) improved efficiencies were from 48.8% (lignite) to 64.17% and 63.70% respectively. Similar improvements were observed with Pb(II) ions from 63% (lignite) to 84.30% and 84.60% respectively. The analysis of the adsorption isotherms for Cd(II) and Pb(II) show the *R*^2^ to be 0.984 and 0.992 favouring the Langmuir isotherm and thus a monolayer adsorption for both metals. The kinetics of adsorption was better described by pseudo-second order than pseudo-first order model.

## Author contributions

ME organized the research problem on the effect of coal ranks and coal biomass blends on the removal of cadmium and lead from aqueous solution and made some scientific inputs on interpretation of the results, and participated in writing the manuscript. OO conducted the experimental work on the preparation of the adsorbent and batch adsorption tests, and participated in writing the manuscript. JA conceived theoretical inputs on process parameters affecting batch adsorption of cadmium (II) and lead (II) ions from aqueous solution, and participated in writing the manuscript.

### Conflict of interest statement

The authors declare that the research was conducted in the absence of any commercial or financial relationships that could be construed as a potential conflict of interest.
